# Impact of Frailty on the Outcomes of Patients with Pancreatic Cancer Undergoing Neoadjuvant Therapy

**DOI:** 10.3390/cancers17244030

**Published:** 2025-12-18

**Authors:** Nicholas R. Williams, Thomas Leuschner, Amanda K. Walsh, Kayla Gault, Amber Ingram, Alex B. Blair, Susan Tsai, Timothy M. Pawlik, Mary E. Dillhoff, Jordan M. Cloyd

**Affiliations:** Department of Surgery, Division of Surgical Oncology, The Ohio State University Wexner Medical Center, Columbus, OH 43210, USA; nicholas.williams@osumc.edu (N.R.W.);

**Keywords:** pancreatic cancer, neoadjuvant therapy, frailty

## Abstract

**Background**: Neoadjuvant therapy (NT) is increasingly utilized for patients with localized pancreatic ductal adenocarcinoma (PDAC). Toxicities during NT are common, often leading to the inability to undergo surgical resection, yet risk factors for attrition are poorly understood. Therefore, we sought to evaluate the impact of baseline frailty on outcomes of patients with PDAC undergoing NT. **Methods**: All patients with potentially resectable (PR) or borderline resectable (BR) PDAC who initiated neoadjuvant chemotherapy and/or chemoradiation between 2019 and 2025 at a single institution were assessed retrospectively in an intention-to-treat fashion. The association between frailty as defined by the modified 11-item frailty index (mFI-11) and receipt of surgical resection as well as other secondary endpoints was assessed. Comprehensive functional frailty assessments were prospectively obtained in a subset of patients. **Results**: Among 252 eligible patients, the median age was 67 years, 56.7% were male, 90.9% were White, 49.6% had PR disease, and 5.2% were frail according to mFI-11. After a median 3.6 months of NT, 62.7% underwent surgical resection. Frail individuals had worse performance status and increased comorbidities compared with non-frail patients. On multivariable analysis, male sex, BR anatomic staging, initial use of Gemcitabine + nab-paclitaxel, and frailty (OR 0.09; 95%CI 0.02–0.44) were associated with reduced odds of undergoing resection. Along with increased baseline CA 19-9 levels, frailty was independently associated with worse overall survival (HR 3.00; 95%CI 1.46–6.20). Among 39 patients who underwent formal functional frailty assessment, only abnormal posture was associated with lower odds of surgical resection following NT (OR, 0.22; 95% CI, 0.05–0.92), and no aspects of functional frailty were associated with overall survival. **Conclusions**: Among patients with localized PDAC initiating NT, frailty as assessed by mFI-11 was associated with reduced odds of undergoing surgical resection and worse overall survival. Future research should focus on efforts to improve functional status during NT.

## Simple Summary

Pancreatic cancer is a common cause of cancer-related death and carries an exceedingly poor prognosis. Increasingly, patients with localized disease are receiving chemotherapy and/or radiation therapy prior to surgical resection, a treatment plan known as neoadjuvant therapy (NT). Unfortunately, a significant number of patients who initiate NT will be unable to undergo surgery, and it remains unclear which patients are at risk of not completing NT. In this retrospective intention-to-treat cohort study, patient frailty was independently associated with a decreased likelihood of surgical resection and worse overall survival. These findings suggest that frail patients may benefit from different treatment sequencing or from receiving personalized care aimed at strengthening their overall health early in treatment.

## 1. Introduction

Pancreatic ductal adenocarcinoma (PDAC) is the third most common cause of cancer-related death in the United States and has a poor 5-year overall survival (OS) rate of approximately 13% [1]. Traditionally, patients with localized PDAC have undergone surgical resection followed by adjuvant chemotherapy. However, over the past several decades, there has been a steady shift toward the use of neoadjuvant therapy (NT) prior to surgery [2]. Advantages of NT include potential tumor downstaging, improved likelihood of margin-negative resection, the opportunity to treat micrometastatic disease that initial surgery would fail to address, and ensuring the receipt of systemic therapy, which can be difficult to deliver postoperatively due to surgical complications, reduced performance status, or early recurrence [3,4].

Despite these advantages, there are several downsides to administering NT. Treatment-associated adverse events are common, and severe toxicities can occur which can lead to delays or interruptions to treatment as well as functional decline [5,6]. At the same time, challenges associated with interdisciplinary patient care coordination persist [7], and the optimal treatment regimens and duration have yet to be determined [8]. All of these factors complicate the delivery of NT and increase the potential for patients to not ultimately undergo surgical resection, which is associated with worse OS outcomes [9,10]. Nevertheless, risk factors for surgical attrition are poorly understood [10].

Frailty can be understood as a geriatric syndrome characterized by an increased vulnerability to adverse health outcomes, arising from the cumulative effect of multiple health deficits and a diminished capacity to respond to physiological stressors [11]. Frailty is common among patients with PDAC and is associated with worse long-term outcomes [12,13]. Cancer patients who are frail experience higher rates of postoperative complications, worse responses to chemotherapy, and reduced OS [14,15]. Despite these realities, the impact of frailty on patient outcomes during NT is unknown. A better understanding of the outcomes of frail patients could identify opportunities to develop patient-centered interventions to support physiological needs during NT and/or clarify alternative treatment sequencing.

## 2. Materials and Methods

### 2.1. Overall Study Design

The study was designed to evaluate the impact of baseline frailty on outcomes of patients with localized PDAC undergoing NT. In this retrospective intention-to-treat cohort study, all patients with potentially resectable (PR) or borderline resectable (BR) PDAC who received at least one cycle of NT with the intent of undergoing surgical resection were included. Patients were seen at either the Ohio State University Comprehensive Cancer Center’s Pancreatic Cancer Multidisciplinary Clinic (PMDC) or in a physician’s own clinic. The study was approved by the Ohio State University Institutional Review Board. Patients diagnosed between November 2018 and January 2025 were included, with a data cut-off date of 31 August 2025. Patients were excluded from this study if they were diagnosed with locally advanced (LA) or metastatic cancer at presentation, if comorbidities were deemed to be prohibitive of subsequent surgical resection, or if upfront surgery was selected.

The modified 11-item frailty index (mFI-11) was used to assess the baseline frailty of all patients included in the study [16]. The specific conditions included in the mFI-11 are a history of diabetes mellitus, non-independent functional status, chronic obstructive pulmonary disease, congestive heart failure, myocardial infarction, past percutaneous coronary intervention/cardiac surgery/angina, hypertension requiring the use of medication, peripheral vascular disease, impaired sensorium, transient ischemic attack/cerebrovascular accident without deficit, and cerebrovascular accident with deficit. Each comorbidity or deficit was coded as 1 if present and 0 if absent. These values were summed, then divided by the total number of variables considered, 11, yielding a score between 0 and 1 for each patient, with higher scores reflecting increased frailty. Frailty was defined as a score of 0.55 or greater [17].

### 2.2. Comprehensive Frailty Assessments

A subset of patients evaluated in the PMDC underwent comprehensive frailty evaluation by trained physical therapists (PT). The indications for a patient to receive a frailty assessment included the presence of baseline neuropathy, Eastern Cooperative Oncology Group Performance Status (ECOG PS) ≥ 1, self-reported sedentary lifestyle, or verbalized concerns regarding their ability to tolerate NT or surgery. Patients were retrospectively categorized as either frail or not frail based on the Fried frailty phenotype [18]. This widely used classification system proposes the five domains of exhaustion, grip strength, gait speed, weight loss, and physical activity as necessary to assess functional frailty. Abnormalities in three or more of these areas are sufficient for a frailty designation. Exhaustion was verbally assessed using the scale from the Common Terminology Criteria for Adverse Events (CTCAE v5.0). A patient was considered to be experiencing exhaustion if they admitted “fatigue that is not relieved by rest” (fatigue ≥ Grade 2 on CTCAE). Grip strength was assessed with a Jamar hydraulic hand dynamometer and was identified as abnormal if the patient’s stronger (left vs. right) measure fell below established BMI and sex-matched cutoffs [19]. Gait speed was assessed via a timed 3-m walk, with any time-to-complete greater than 3.62 s considered to be abnormal. Physical activity status was assessed retrospectively based on the PT’s subjective assessment describing level of functioning, ability to complete activities of daily living, participation in recreational activities, and mobility limitations. Weight loss was defined as a loss of 10 lbs. or more in the prior 12 months.

### 2.3. Statistical Analysis

The primary aim of the study was to evaluate baseline patient frailty as a potential predictor of the ability to undergo surgical resection following NT completion. Continuous variables were first evaluated for normality using the Shapiro–Wilk test. Normally distributed variables were analyzed with Independent Samples t-tests and reported as means, while non-normally distributed variables were assessed using the non-parametric Mann–Whitney test and presented as medians. Categorical variables were compared using chi-square tests, with Fisher’s Exact tests applied when assumptions for chi-square were not met. To address missing data points prior to regression analysis, we performed multiple imputation, generating five imputed datasets. Results from these datasets were pooled to obtain overall estimates and assess statistical significance. To determine the association of variables with surgical resection status, logistic regression analysis was performed. First, univariate analyses were conducted to assess associations between baseline frailty, as well as clinical, demographic, and oncologic determinants of health, and performance of surgical resection. All variables with *p* < 0.10 on univariate analysis were entered into a multivariable logistic regression to identify factors independently associated with surgical resection status. Similarly, to assess the relationship of factors with OS, univariate Cox regression analyses were performed, with *p* < 0.10 on univariate analysis prompting factors to be considered for Cox multivariable regression analysis. For both multivariable logistic regression and multivariable Cox regression analyses, age, sex, and anatomic staging were included in the analysis, regardless of their univariate significance. All statistical analyses were performed using IBM SPSS (version 29.0) with statistical significance set at *p* < 0.05 (IBM Corp., Armonk, NY, USA).

## 3. Results

### 3.1. Patient Characteristics

Among the 252 patients who met the inclusion criteria, the median age was 67 years, 56.7% were male, and 90.9% were White. A similar proportion had PR (49.6%) and BR (50.4%) disease, and most patients (65.9%) received FOLFIRINOX as their initial chemotherapy, with 24.6% also receiving chemoradiation. Based on mFI-11, 13 patients (5.2%) were considered frail, while 239 (94.8%) were not. Table 1 compares the clinical and demographic characteristics of frail and non-frail patients. Frail patients had a higher Charlson Comorbidity Index score than non-frail patients (median (IQR), 9 (3) vs. 5 (2); *p* < 0.001) and were more likely to have an ECOG PS greater than 1 (46.2% vs. 5.0%; *p* < 0.001). Overall, frail patients were less likely to undergo surgical resection (15.4% vs. 65.3%; *p* < 0.001).

### 3.2. Predictors of Resection Status and OS

Median follow-up for the cohort was 46.5 months. Table 2 displays the outcomes of univariate and multivariable logistic regression analysis of factors associated with surgical resection. On multivariable analysis, male sex (OR, 0.50; 95% CI, 0.29–0.89), frailty (OR, 0.09; 95% CI, 0.02–0.44), BR stage (OR, 0.42; 95% CI, 0.24–0.73), and initial use of Gemcitabine + nab-paclitaxel during NT (OR, 0.43; 95% CI, 0.22–0.84) were independently associated with decreased odds of resection.

**Figure 1:**
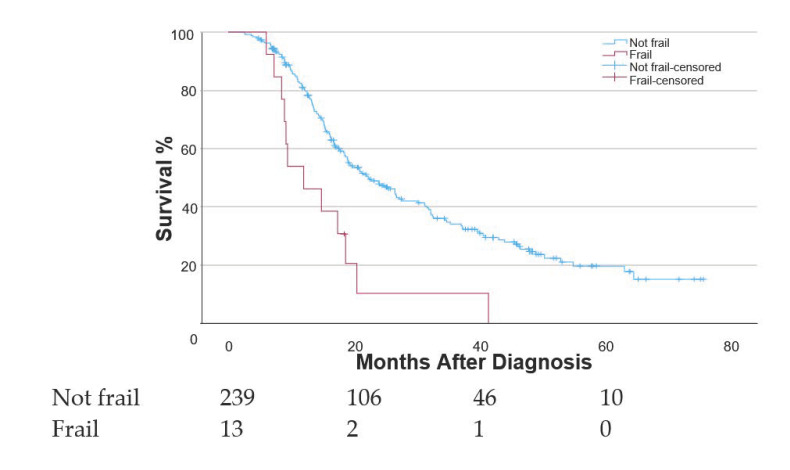
Kaplan–Meier curve displaying overall survival measured from the time of diagnosis among patients who had frail and non-frail mFI-11 scores.

Compared to frail patients, non-frail patients experienced superior median OS (22.3 [95% CI 17.60–27.06] vs. 12.1 [95% CI 5.42–18.72] months) and improved 2-year OS (48.4% vs. 10.3%) (Figure 1). On multivariable Cox regression, frailty (HR, 3.00; 95% CI, 1.46–6.20), increased baseline CA 19-9 levels (HR, 1.00; 95% CI, 1.00–1.01), ECOG PS (HR, 1.37; 95% CI, 0.98–1.91), and use of Gemcitabine + nab-paclitaxel (HR, 2.19; 95% CI, 1.43–3.33) were associated with worse OS (Table 3).

### 3.3. Functional Frailty Analysis

Of the 252 patients initially included in this study, 39 underwent comprehensive frailty assessment before beginning NT. Among this subset, 23 (59.0%) were functionally frail according to Fried criteria. No association was observed between functional frailty and subsequent surgical resection; however, functional frailty was associated with significant differences in albumin levels, ECOG PS scores, and initial neoadjuvant chemotherapy regimen compared to non-frail patients (Table S1). When each aspect of the functional frailty assessment was considered in a univariate logistic regression analysis, only the finding of abnormal patient posture (OR, 0.22; 95% CI, 0.05–0.92) was associated with surgical resection. This association persisted even after controlling for age, sex, anatomic stage, and CCI score (OR, 0.08; 95% CI, 0.01–0.68) (Table S2). No aspects of functional frailty were associated with OS on Cox regression analysis.

## 4. Discussion

Contemporary management of localized PDAC consists of multimodality therapy. Although the preferred sequencing remains controversial, NT is increasingly being recommended for most patients with localized PDAC [20,21]. While achieving surgical resection remains prognostically important and a patient-centered endpoint, risk factors for surgical attrition remain poorly understood [10]. In this single-institution retrospective but intention-to-treat study, we found that frail patients, as assessed by the mFI-11, are less likely than their non-frail counterparts to eventually undergo surgical resection. Beyond this association, patient frailty also proved to be an independent predictor of OS. These findings carry important implications for treatment decision-making and utilization of patient-centered resources during cancer therapy.

While frailty has been the center of extensive research in the oncology literature, relatively little research has been conducted on the impact of frailty during NT for PDAC. In addition, most prospective clinical trials limit inclusion to those with good PS [22,23,24]. For example, Fong et al. investigated ECOG PS, which captures some elements of functional frailty, and noted that a drop in PS during NT is independently associated with decreased odds of eventual surgical exploration in patients with PDAC [25]. On the other hand, Dickey et al. reported that baseline ECOG PS alone was not associated with attrition following NT [26].

Furthermore, Funamizu et al. reported that frail patients are less likely to complete adjuvant therapy following pancreatic cancer resection and demonstrate shorter OS [27]. On the other hand, frailty has been assessed in patients undergoing NT for other cancers, such as muscle-invasive bladder cancer, in which frailty was associated with worse OS [28]. Additionally, metrics that capture elements of frailty, such as total number of comorbidities and skeletal muscle wasting during NT, are associated with decreased odds of resection and decreased survival in other cancers, respectively [29,30].

The impact of frailty on the outcomes of patients undergoing NT is important, but whether this represents a modifiable risk factor that can be intervened upon remains uncertain. Given the retrospective nature of our study and the fragmented care that many patients at our institution receive, we were unable to assess the frequency with which frail patients received formal physical therapy or participated in formal prehabilitation programs and whether this improved their functional status during NT. While prehabilitation is a promising strategy for improving patient condition before major surgery, only limited evidence exists for its incorporation into the neoadjuvant setting [31,32,33]. Therefore, additional research on targeted interventions in this patient population is warranted. Alternate treatment sequencing might be considered for those patients with borderline (i.e., upfront surgical resection) or prohibitive (i.e., definitive chemotherapy and/or chemoradiation) frailty status. Finally, prioritizing less invasive surgical approaches may be of particular benefit to frail patients as well [34].

Frailty is a clinical syndrome with a wide variety of features often present in patients with PDAC. One lens through which frailty can be viewed is a cumulative deficit model. This model, as represented by the mFI-11, suggests that the impact of individual comorbidities, past medical events, and other physical deficits can be summed together to accurately depict one’s state of frailty [16]. Alternatively, the phenotype model aims to capture the functional manifestations of frailty, including weakness, slowness, weight loss, exhaustion, and decreased physical activity, as exemplified by the Fried Frailty Index [18].

A strength of our study is the use of both a widely available calculated frailty index as well as comprehensive formal frailty assessments in a subset of patients. On the other hand, the selection of mFI-11 and the use of a relatively high threshold to define frailty may have influenced our study findings. Previous studies have reported higher rates of frailty among patients with PDAC using other frailty indices such as the 5-item mFI, the Charlson Comorbidity Index, and the Fried Frailty Index [12,35,36]. It is also important to recognize that only patients who were deemed potentially candidates for eventual surgical resection were included in our study. Thus, other studies with less stringent inclusion criteria may report higher rates of frailty. Nevertheless, future research on the optimal frailty index for predicting outcomes of NT and informing patient-centered decision making is warranted.

Most patients in the more recent era of our PMDC underwent a comprehensive functional frailty assessment encompassing measures of exhaustion, pain, balance, posture, gait, sit-to-stand performance, grip strength, independence in activities of daily living, and fall history, among other domains. Interestingly, normal posture was the only metric found to be significantly associated with receipt of surgical resection. While these results could be related to the smaller sample size of this subset analysis, one possible interpretation of this finding is that abnormal sitting posture at rest is a potentially valuable indicator of poor functional health and is able to successfully predict a patient’s ability to tolerate NT. On the other hand, these results question whether formal frailty assessments, which are resource-intensive, provide additional value beyond standard clinical assessment. Future investigation into the prognostic value of these metrics with larger groups of patients is necessary.

Whereas strengths of our study include its intention-to-treat nature, where all patients who initiated NT were included, and the use of both a widely available mFI-11 and comprehensive functional frailty assessments, the primary limitation is its retrospective single-institution design. Importantly, both measured and unmeasured differences could exist between frail and non-frail patients (e.g., clinical judgments regarding patient fitness that influence treatment decision making) that confound our results. In addition, several important measures during NT that could influence surgical attrition were not available in our study data, such as chemotherapy dose intensity and toxicity profiles. Functional frailty assessments were also only obtained at baseline. Conducting multiple functional frailty assessments over the course of NT could characterize the importance of an individual’s trend in frailty and potentially identify critical time points when targeted interventions could be most beneficial. Finally, functional assessments were performed in only a small subset, and these patients are probably not representative of the entire cohort, as they were often referred due to concerns regarding their ability to tolerate NT. Indeed, of those classified as non-frail by Fried criteria, most still had deficiencies in at least one assessment (i.e., pre-frail).

## 5. Conclusions

Among patients with PR or BR PDAC undergoing NT, frailty as measured by the mFI-11 was independently associated with a decreased likelihood of surgical resection and worse OS. Frail patients may benefit from alternative treatment sequencing or individualized patient-centered interventions designed to improve physiologic resilience in the early stages of treatment. Future research with larger cohorts is warranted to clarify our findings on the relationship between functional frailty during NT and surgical outcomes.

## Figures and Tables

**Table 1:** Patient Clinical and Demographic Characteristics Based on Baseline mFI-11 Frailty Status.

Characteristic	Not Frail, *n* = 239	Frail, *n* = 13	*p* Value	Missing, *n*
Sex, *n* (%)			0.72	
Female	104 (43.5)	5 (38.5)		
Male	135 (56.5)	8 (61.5)		
Age, y, median (IQR)	67 (13)	63 (17)	0.89	
Race, *n* (%)			0.34 ^a^	
White	218 (91.2)	11 (84.6)		
Black/Unknown	21 (8.8)	2 (15.4)		
BMI, kg/m^2^, median (IQR)	25.16 (7.67)	27.37 (11.71)	0.11	3
Charlson Comorbidity Index, median (IQR)	5 (2)	9 (3)	<0.001	
Albumin, g/dL, median (IQR)	4.0 (0.7)	3.9 (0.9)	0.23	6
ECOG performance status, *n* (%)			<0.001	15
0	93 (38.9)	3 (23.1)		
1	119 (49.8)	4 (30.8)		
≥2	12 (5.0)	6 (46.2)		
Anatomic stage, *n* (%)			0.80	
Potentially resectable	119 (49.8)	6 (46.2)		
Borderline resectable	120 (50.2)	7 (53.8)		
Initial neoadjuvant chemotherapy, *n* (%)			0.03 ^a^	
FOLFIRINOX	160 (66.9)	6 (46.2)		
Gemcitabine + nab-paclitaxel	69 (28.9)	4 (30.8)		
Other	10 (4.2)	3 (23.1)		
Days from diagnosis to NT, median (IQR)	20 (13)	18 (11)	0.90	7
Total days spent in NT, median (IQR)	108 (89)	166 (119)	0.12	17
ER visit or hospital admission during NT, *n* (%)			0.06 ^a^	2
No	139 (58.2)	3 (23.1)		
Yes	100 (41.8)	8 (61.5)		
Chemotherapy dose reduction, *n* (%)			0.84	17
No	109 (45.6)	5 (38.5)		
Yes	115 (48.1)	6 (46.2)		
Surgical resection, *n* (%)			<0.001 ^a^	
No	83 (34.7)	11 (84.6)		
Yes	156 (65.3)	2 (15.4)		
Pre-NT CA 19-9, U/mL, median (IQR)	191.1 (753.8)	1274.1 (2651.3)	0.08	5
Post-NT CA 19-9, U/mL, median (IQR)	45.3 (206.8)	82.8 (2166.7)	0.18	14

Abbreviations: BMI, body mass index; ECOG, Eastern Cooperative Oncology Group; NT, neoadjuvant therapy. ^a^ Fisher’s Exact Test.

**Table 2:** Univariate and Multivariable Logistic Regression of Factors Associated with Surgical Resection.

	Univariate		Multivariable	
Characteristic	OR (95% CI)	*p* Value	OR (95% CI)	*p* Value
Sex				
Female	Ref		Ref	
Male	0.54 (0.32–0.92)	0.02	0.50 (0.29–0.89)	0.02
Age	0.98 (0.96–1.01)	0.25		
Race				
White	Ref			
Black/Unknown	0.62 (0.26–1.47)	0.28		
BMI	0.99 (0.95–1.04)	0.76		
Modified 11-Item Frailty Index				
<0.55	Ref		Ref	
≥0.55	0.10 (0.02–0.45)	0.003	0.09 (0.02–0.44)	0.003
Albumin	1.40 (0.87–2.23)	0.17		
ECOG performance status		0.21		
0	Ref			
1	0.68 (0.39–1.19)	0.18		
≥2	0.49 (0.19–1.30)	0.15		
Anatomic stage				
Potentially resectable	Ref		Ref	
Borderline resectable	0.48 (0.29–0.81)	0.01	0.42 (0.24–0.73)	0.002
Pre-NT CA 19-9	1.00 (0.99–1.00)	0.28		
Initial neoadjuvant chemotherapy		0.04		0.04
FOLFIRINOX	Ref		Ref	
Gemcitabine + nab-paclitaxel	0.48 (0.28–0.85)	0.01	0.43 (0.22–0.84)	0.01
Other	0.75 (0.23–2.40)	0.63	0.98 (0.24–4.04)	0.98

Abbreviations: BMI, body mass index; ECOG, Eastern Cooperative Oncology Group; NT, neoadjuvant therapy; Ref, reference.

**Table 3:** Cox Regression Analysis of Factors Associated with Overall Survival.

	Univariate		Multivariable	
Characteristic	HR (95% CI)	*p* Value	HR (95% CI)	*p* Value
Sex				
Female	Ref			
Male	1.14 (0.83–1.55)	0.42		
Age	1.01 (1.00–1.03)	0.13		
Race				
White	Ref			
Black/Unknown	1.43 (0.84–2.45)	0.19		
BMI	0.99 (0.97–1.02)	0.49		
Modified 11-Item Frailty Index				
<0.55	Ref		Ref	
≥0.55	2.64 (1.46–4.77)	0.001	3.00 (1.46–6.20)	0.003
Albumin	0.89 (0.67–1.15)	0.34		
ECOG performance status		0.06		0.02
0	Ref		Ref	
1	1.44 (1.03–2.00)	0.03	1.37 (0.98–1.91)	0.06
≥2	0.91 (0.46–1.82)	0.79	0.60 (0.27–1.34)	0.21
Anatomic stage				
Potentially resectable	Ref			
Borderline resectable	1.29 (0.95–1.76)	0.10		
Pre-NT CA 19-9	1.00 (1.00–1.01)	0.01	1.00 (1.00–1.01)	0.02
Initial neoadjuvant chemotherapy		<0.001		<0.001
FOLFIRINOX	Ref		Ref	
Gemcitabine + nab-paclitaxel	1.88 (1.35–2.63)	<0.001	2.19 (1.43–3.33)	<0.001
Other	1.47 (0.71–3.02)	0.30	1.06 (0.45–2.49)	0.90

Abbreviations: BMI, body mass index; ECOG, Eastern Cooperative Oncology Group; NT, neoadjuvant therapy; Ref, reference.

## Data Availability

Research data are stored in an institutional repository and will be shared upon request to the corresponding author.

## Supplementary Materials

The following supporting information can be downloaded at: https://www.mdpi.com/article/10.3390/cancers17244030/s1. Table S1: Patient Clinical and Demographic Characteristics Based on Baseline Fried Frailty Classification; Table S2: Univariate and Multivariable Logistic Regression of Factors Associated with Surgical Resection.
