# Supplementary material for: Impact of Frailty on the Outcomes of Patients with Pancreatic Cancer Undergoing Neoadjuvant Therapy
Source: Cancers (Basel). 2025 Dec 18;17(24):4030. doi: 10.3390/cancers17244030 (PMC12731661; doi:10.3390/cancers17244030)
Supplement: Supplementary file 1 [file cancers-17-04030-s001.zip › Supplementary Table S1.pdf]

Table S1. Patient Clinical and Demographic Characteristics Based on Baseline Fried Frailty Classification.

Characteristic	Not frail, n=16	Frail, n=23	*P* Value	Missing, n
Sex, n (%)			0.50	
Female	8 (50.0)	14 (60.9)		
Male	8 (50.0)	9 (39.1)		
Age, y, mean (SD)	67.9 (9.1)	72.0 (7.4)	0.12[b]	
Race, n (%)			1.00[a]	
White	13 (81.3)	20 (87.0)		
Black	2 (12.5)	2 (8.7)		
Unknown	1 (6.3)	1 (4.3)		
BMI, kg/m^2, mean (SD)	27.7 (7.3)	29.7 (6.3)	0.36[b]	
Charlson Comorbidity Index, median (IQR)	5 (1.5)	6 (2)	0.05	
Modified 11-Item Frailty Index, n (%)			0.26[a]	
<0.55	16 (100.0)	20 (87.0)		
≥0.55	0 (0.0)	3 (13.0)		
Albumin, g/dL, median (IQR)	4.2 (0.4)	3.9 (0.6)	0.01	1
ECOG performance status, n (%)			0.01[a]	
0	2 (12.5)	0 (0.0)		
1	14 (87.5)	15 (65.2)		
≥2	0 (0.0)	8 (34.8)		
Anatomic stage, n (%)			0.24	
Potentially resectable	6 (37.5)	13 (56.5)		
Borderline resectable	10 (62.5)	10 (43.5)		
Initial neoadjuvant chemotherapy, n (%)			0.02[a]	
FOLFIRINOX	12 (75.0)	7 (30.4)		
Gemcitabine + nab-paclitaxel	3 (18.8)	12 (52.2)		
Other	1 (6.3)	4 (17.4)		
Neoadjuvant radiation, n (%)			1.00[a]	
No	12 (75.0)	18 (78.3)		
Yes	4 (25.0)	5 (21.7)		
Days from diagnosis to NT, median (IQR)	26 (9.5)	17 (15.0)	0.28	2
Total days spent in NT, median (IQR)	121 (72.0)	123 (91.0)	0.57	5
ER visit or hospital admission during NT, n (%)			0.29	
No	9 (56.3)	9 (39.1)		
Yes	7 (43.8)	14 (60.9)		
Completed NT as planned, n (%)			0.38	5
No	7 (43.8)	13 (56.5)		
Yes	7 (43.8)	7 (30.4)		
Surgical resection, n (%)			0.37	
No	6 (37.5)	12 (52.2)		
Yes	10 (62.5)	11 (47.8)		
Pre-NT CA 19-9, U/mL, median (IQR)	598.99 (1261)	233.63 (627)	0.22	
Post-NT CA 19-9, U/mL, median (IQR)	144.59 (457)	46.88 (118)	0.10	3

Abbreviations: BMI, body mass index; ECOG, Eastern Cooperative Oncology Group; NT, neoadjuvant therapy.

[a]Fisher's Exact Test

[b]Independent Samples T-Test
