# Supplementary material for: Impact of Frailty on the Outcomes of Patients with Pancreatic Cancer Undergoing Neoadjuvant Therapy
Source: Cancers (Basel). 2025 Dec 18;17(24):4030. doi: 10.3390/cancers17244030 (PMC12731661; doi:10.3390/cancers17244030)
Supplement: Supplementary file 1 [file cancers-17-04030-s001.zip › Supplementary Table S2.pdf]

Table S2. Univariate and Multivariable Logistic Regression of Factors Associated with Surgical Resection.

	Univariate		Multivariable	
**Characteristic**	**OR (95% CI)**	***P* Value**	**OR (95% CI)**	***P* Value**
Sex				
Female	Ref			
Male	1.43 (0.40-5.12)	0.58		
Age	1.01 (0.94-1.10)	0.72		
Race				
White	Ref			
Black/Unknown	0.37 (0.06-2.30)	0.29		
BMI	0.93 (0.84-1.02)	0.14		
Modified 11-Item Frailty				
0-5	Ref			
≥6	0.00 (0.00)	1.00		
Albumin	1.06 (0.26-4.26)	0.94		
ECOG performance status		1.00		
0	Ref			
1	0.00 (0.00)	1.00		
≥2	0.00 (0.00)	1.00		
Anatomic stage				
Potentially resectable	Ref			
Borderline resectable	0.48 (0.13-1.72)	0.26		
Fried frailty classification				
Not frail	Ref			
Frail	0.55 (0.15-2.02)	0.37		
DVPRS (at rest)	0.93 (0.73-1.18)	0.54		
Exhaustion				
Normal	Ref			
Abnormal	1.00 (0.26-3.80)	1.00		
Gait speed				
Normal	0.00 (0.00)	1.00		
Abnormal	Ref			
Physical activity status				
Active	Ref			
Inactive	0.77 (0.21-2.77)	0.69		
Grip Strength				
Normal	Ref			
Abnormal	0.35 (0.09-1.34)	0.13		
Weight loss (10lb in 12m)				
Present	0.85 (0.16-4.43)	0.85		
Absent	Ref			
Sit to Stand Independence				
No	0.57 (0.10-3.41)	0.54		
Yes	Ref			
Gait appearance				
Normal	Ref			
Abnormal	0.79 (0.16-3.91)	0.78		
Posture				
Normal	Ref			
Abnormal	0.22 (0.05-0.92)	0.04	0.08 (0.01-0.68)	0.02
Fall within last year				
No	Ref			
Yes	0.62 (0.17-2.21)	0.46		
SPPB Score		0.99		
0-6	Ref			
7-9	0.93 (0.24-3.64)	0.92		

10-12	0.00 (0.00)	1.00
5x sit to stand test time	1.04 (0.96-1.13)	0.30
"Up and Go Test" time	1.04 (0.95-1.14)	0.39

Abbreviations: BMI, body mass index; ECOG, Eastern Cooperative Oncology Group; NT, neoadjuvant therapy; DVPRS, Defense and Veterans Pain Rating Scale; SPPB, Short Physical Performance Battery; Ref, reference.
